# Response to letter by Saumarez *et al*. entitled ‘Regarding the editorial by Sau and Ng. “Hypertrophic cardiomyopathy risk stratification based on clinical or dynamic electrophysiological features: two sides of the same coin”’

**DOI:** 10.1093/europace/euad174

**Published:** 2023-06-20

**Authors:** Arunashis Sau, Fu Siong Ng

**Affiliations:** National Heart and Lung Institute, Imperial College London, Hammersmith Campus, Du Cane Road, London W12 0NN, UK; Department of Cardiology, Imperial College Healthcare NHS Trust, London W12 0HS, UK; National Heart and Lung Institute, Imperial College London, Hammersmith Campus, Du Cane Road, London W12 0NN, UK; Department of Cardiology, Imperial College Healthcare NHS Trust, London W12 0HS, UK; Department of Cardiology, Chelsea and Westminster Hospital NHS Foundation Trust, London SW10 9NH, UK

**This is a response to the Letter to the Editor ‘Regarding the editorial by Sau and Ng. “Hypertrophic cardiomyopathy risk stratification based on clinical or dynamic electrophysiological features: two sides of the same coin”’ by Saumarez *et al*. https://doi.org/10.1093/europace/euad175, about the article ‘Hypertrophic cardiomyopathy risk stratification based on clinical or dynamic electrophysiological features: two sides of the same coin’ by A.S. and F.S.N https://doi.org/10.1093/europace/euad072.**

We thank Saumarez *et al.* for their letter discussing our editorial.^1^ We fully agree that current risk stratification in hypertrophic cardiomyopathy (HCM) is limited in accuracy and we must aspire to better. Any effort to improve risk stratification for this important group of patients, including the work of Saumarez and colleagues, is to be commended. As electrophysiologists, we are pleased that there are clear efforts to use the electrophysiological (EP) substrate to guide risk stratification.

As discussed in our editorial, paced electrogram fractionation analysis (PEFA) is an interesting potential improvement on the current paradigm. However, the current data supporting its use are limited to small studies. The current evidence base is somewhat analogous in size to the initial descriptions of EP testing for ventricular tachycardia (VT) inducibility for risk stratification in Brugada syndrome. Initial reports suggested that VT inducibility was highly predictive for arrhythmic events.^2^ However, the subsequent data in external cohorts^3^ failed to confirm the initially high predictive values, and therefore the role of VT stimulation in Brugada syndrome is currently relatively limited.^4^

We therefore feel that, although there is certainly a large gap in current risk stratification approaches in HCM, there remain unanswered questions before PEFA can be recommended. We encourage Saumarez *et al*., and others, to perform larger scale external validation of invasive EP testing in HCM. Furthermore, an assessment of the reproducibility of the technique on repeated measures would be valuable. Further data such as these may persuade clinicians to adopt invasive EP testing for HCM into routine clinical practice and may increase the chances of its incorporation into clinical guidelines. As it stands, as alluded to in our original editorial,^1^ PEFA is possibly a useful adjunct, but because of the relatively small studies on which it is based, it is not currently ready to replace the existing, albeit flawed, paradigm of HCM risk stratification.

Our concerns around the use of invasive EP testing for risk stratification of HCM are also related to a small, but ever-present, risk from an invasive procedure. This is particularly important given the very large numbers of these procedures that would need to be performed on asymptomatic relatives, if current guidelines were to be replaced.

The ideal solution to address the flawed current paradigm, and the challenge of invasive risk stratification and need for repeated measures over time, is a simple, non-invasive tool such as body surface potential mapping or the electrocardiogram (ECG). Our view is that research efforts should be targeted towards these non-invasive approaches, which may be equally capable of capturing the dynamic EP substrate. An additional advantage is that the body surface potential mapping or the ECG can capture information from the whole heart rather than being limited to the right ventricle, which is often the case in invasive EP testing. The recent rapid developments in artificial intelligence applied to surface ECG signals may mean that the subtle signatures of risk within the ECG may be used in the near future to aid risk stratification in HCM.

## Data Availability

Data availability not applicable to this article as no datasets were generated or analysed.
